# Prevalence of an incompetent lip seal during growth periods throughout Japan: a large-scale, survey-based, cross-sectional study

**DOI:** 10.1186/s12199-021-00933-5

**Published:** 2021-01-21

**Authors:** Yukiko Nogami, Issei Saitoh, Emi Inada, Daisuke Murakami, Yoko Iwase, Naoko Kubota, Yuki Nakamura, Masami Kimi, Haruaki Hayasaki, Youichi Yamasaki, Yasutaka Kaihara

**Affiliations:** 1 Division of Pediatric Dentistry, Graduate School of Medical and Dental Science, Niigata University, 2-5274 Gakkocho-dori, Chuo-ku, Niigata, Japan; 2 Department of Pediatric Dentistry, Kagoshima University Graduate School of Medical and Dental Sciences, 8-35-1 Sakuragaoka, Kagoshima, Japan; 3 Kimi Dental and Oral Clinic, 122-1 Aza Ishidaka Oaza Kuroishi Aizuwakamatsu, Fukushima, Japan; 4 Department of Dental Hygiene, Ogaki Women’s College, 1-109 Nishinokawa-cho, Ogaki, Gifu, Japan

**Keywords:** Incompetent lip seal, Abnormal oral habits, Epidemiology, Japanese children, Orofacial morphology, Mouth breathing

## Abstract

**Background:**

Systemic and local factors may lead to disruption of craniofacial growth and development, causing an imbalance between the orofacial skeleton, muscle and soft tissue, dental occlusion, and the dental arch during growth periods. We aimed to reveal whether the prevalence of incompetent lip seal (ILS) varies with age and region, as well as to clarify the factors related to an ILS, in a national, large-scale epidemiological study.

**Methods:**

We surveyed 3399 children, from 3 to 12 years of age, visiting 66 pediatric dental clinics throughout Japan. For this survey, we employed a questionnaire consisting of 44 questions regarding daily health conditions and lifestyle habits. We evaluated the differences in ILS prevalence by age and region (using a Cochran-Armitage test for trend and a Kruskal-Wallis test), and the relationship between ILS and factors investigated in the questionnaire (using Spearman’s rank correlation coefficient).

**Results:**

We observed that 30.7% of Japanese children exhibited an ILS and that the ILS rate increased with age (*p* < 0.001). There were no regional differences in the rate of ILS in Japanese children (*p* = 0.506). We revealed that 12 of 44 survey items exhibited a statistically significant correlation with ILS (*p* < 0.001), using Spearman’s rank correlation coefficient. These items involved orofacial morphology, mouth breathing, and possibly, allergic rhinitis.

**Conclusion:**

The rate of ILS seems to increase with age in children, throughout Japan. Therefore, this disorder may not self-correct during the growth periods in these children. Guidelines are required for pediatric dentists to recognize ILS among children aged 3–12 years.

**Supplementary Information:**

The online version contains supplementary material available at 10.1186/s12199-021-00933-5.

## Background

It is well known that abnormal oral habits, such as unusual speech, abnormal swallowing, tongue dysfunction [1–3], an incompetent lip seal (ILS), mouth breathing, and bad dietary habits, have serious consequences on the healthy development of oral function during growth periods [4–13]. The entrenchment of an ILS, especially at an early stage, may result in prolonged abnormal oral habits, which may disturb the healthy development of oral function in children [14]. An ILS may indicate an altered lip and facial muscle tone, mouth breathing, vertical and/or sagittal facial discrepancies, inadequate lip length, or an increased anterior lower facial height [12, 15, 16]. Oral posture may be related to the occurrence of malocclusion [17]. Oral morphology, function, and posture are closely related and interdependent [1], developing together to increase articulatory precision and coordination for interpersonal communication [18]. As weakening of the lip seal causes an imbalance between lip and tongue pressure, it may exacerbate labioclination of the anterior maxillary teeth and narrowing of the maxillary dental arch. In one study, orthodontic patients with an ILS had a lower lip pressure than those with no particular oral habits [3]. Open mouth posture is associated with a narrow maxillary dental arch and an increased facial height [19, 20]. Thus, there is clinical and experimental evidence for an association between an ILS and malocclusion.

Kogue et al. revealed a difference in ILS-related factors between the presence and absence of mouth breathing in nursery school children [21]. In one study, a statistically significant correlation was discovered between ILS and nasal area in children exhibiting an ILS more than 80% of the time [22]. Gross et al. demonstrated a positive relationship between an ILS and maxillary arch growth in children [23, 24]. Wagaiyu and Ashley revealed that ILS increased gingival inflammation in schoolchildren aged 11–14 [25]. There have also been cross-sectional studies on the characteristics of American children with an ILS [22, 26]. However, characteristics associated with an ILS in Japanese children have not been elucidated.

In previous studies, 17/63 (27%) Japanese adults with malocclusion [27] and 23/53 (43%) Japanese children (approximately 10 years of age) with malocclusion [28] exhibited an ILS. Gross et al. reported a rate of ILS in 348 children (aged 5.8–8.2 years) of almost 48%, with a higher prevalence in boys than in girls [22]. According to a small cross-sectional study, the rate of ILS may decrease with age [24]. The prevalence of ILS may also vary depending on setting conditions, race, and environment [22]. However, to our knowledge, there have been no reports on large-scale surveys evaluating the rate of ILS in childhood during development. Therefore, the purpose of this study was to verify whether the prevalence of ILS varies with age and region and to determine which factors are related to an ILS, in a national, large-scale epidemiological study.

## Methods

### Aims

To verify whether the prevalence of ILS varies with age and region and to determine which factors are related to an ILS.

### Participants

We requested the cooperation of 80 private dental clinics that specialize in pediatric dentistry throughout Japan from 1st August to 31st October 2014. The study participants comprised pre-school and elementary school students aged 3–12 years. Participants with craniofacial malformations owing to chromosomal abnormalities were excluded. Written informed consent was obtained from all participants’ parents or guardians, and the study was approved by the epidemiological ethics committee of our institution (approval number 26-R8-05-18).

### Questionnaire

The parents or guardians of each participant completed a modified version of the questionnaire used in our preliminary research [11] and developed to reveal relevant factors and combination of factors that might affect an ILS in children. The questionnaire included 44 items regarding daily health conditions and lifestyle habits, and all questions were in Japanese (Additional file 1). It was able to evaluate internal reliability, criterion-related validity, and construct validity for items related to ILS in the preliminary study. The reliability and validity of this questionnaire as demonstrated in Cronbach’s alpha was 0.87. Questions were based on a four-point scale (no = 1; do not think so = 2; think so = 3; yes = 4).

### Statistical analysis

The prevalence of scores for each question item was calculated and analyzed for an association with the prevalence of ILS. We defined question item 18, “Is your child’s mouth often open during the day?” as the presence of an ILS in this study. A two-sided Kruskal-Wallis test was used to evaluate the regional differences in question survey items in Japanese children. The relationship between ILS and regional differences was assessed using the chi-squared test for independence. The relationship between the presence of an ILS and question items was analyzed using Spearman’s rank correlation coefficient with Bonferroni correction. Furthermore, the relationship between question item scores and lip sealing status was analyzed using the Cochran-Armitage test for trend. This test can verify whether there is a change in the rate of ILS with age in this sample. For this test, scores of 3 or 4 were classified as the “Yes group,” and scores of 1 or 2 were classified as the “No group.” These data met the assumptions of the tests owing to the existence of a sufficient sample size. In this study, the adjusted relative risk was calculated using the Mantel-Haenszel technique, evaluating the confounding factors by age. This sample, which had a similar variance between groups that were statistically compared, was appropriate to perform statistical analyses. Data were analyzed using IBM SPSS Statistics for Windows, Version 20.0 (IBM Corp., Armonk, NY, USA) and R for Windows (version 3.6.3; R Foundation for Statistical Computing, Vienna, Austria). Statistical significance was set at *p* < 0.05.

## Results

**Fig. 1:**
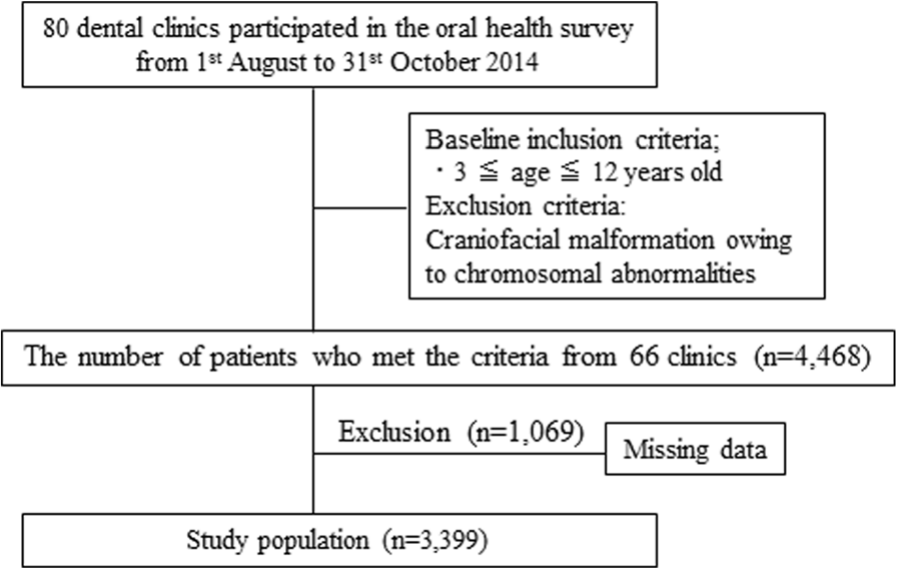
Study design and selection of the study population

**Fig. 2:**
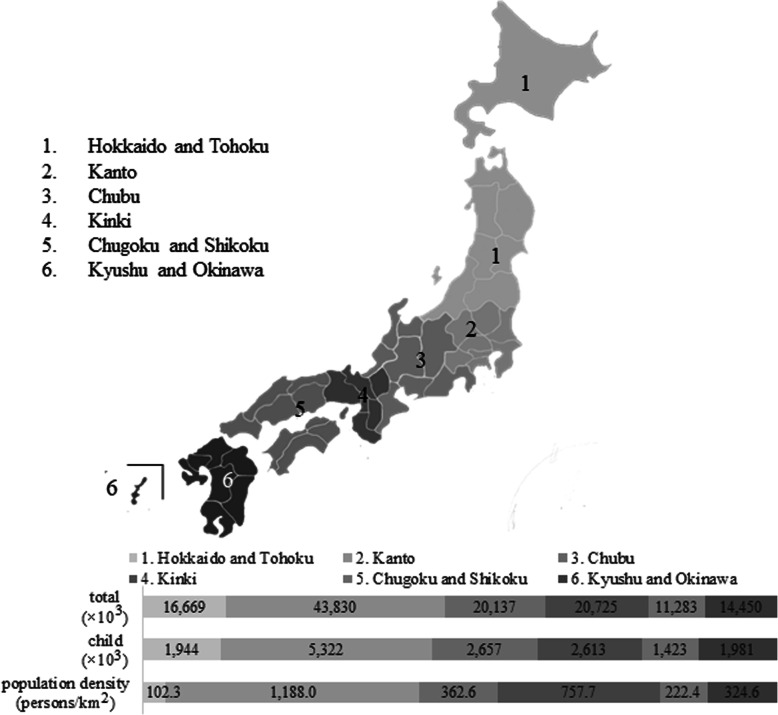
The six regions in Japan and number of people and children in each area, population by 2015 Population Census

**Fig. 3:**
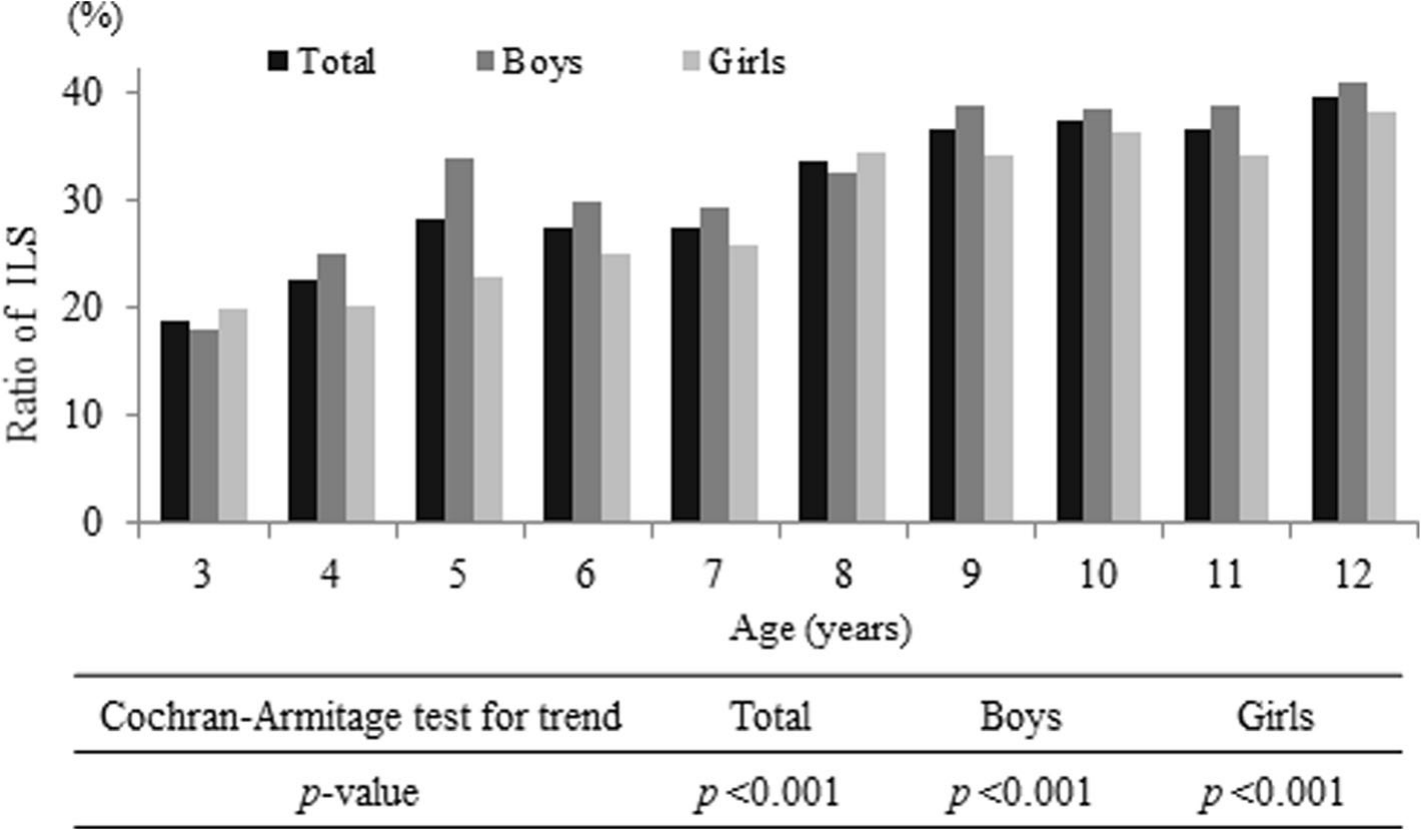
The age-dependent rate of incompetent lip seal (ILS) in Japanese children (%). An incompetent lip seal was defined as an answer of “yes” or “think so” to question item 18—“Is your child’s mouth often open during the day?”

We obtained questionnaires completed in writing by the parents or guardians of 4468 patients (2133 boys and 2335 girls) visiting 66 different pediatric dental clinics. After excluding the incomplete questionnaires, we analyzed the questionnaires of 3399 children (1661 boys and 1738 girls; mean age, 7.8 years’ standard deviation, 2.6 years) (Fig. 1, Table 1). ILS prevalence was stratified by age and six regions in Japan (Fig. 2, Table 2). The distribution of responses to question item 18, which we defined as the question corresponding to the presence of an ILS, is indicated in Table 3. The prevalence of ILS in children based on an answer of “yes” or “think so” to question item 18 (“Is your child’s mouth often open during the day?”) was 30.7% (Table 3). Figure 3 illustrates that the rate of ILS increased with age in Japanese children (*p* < 0.001).

**Table 1:** Number of participants by age

Age		Total	Boys	Girls
3		253 (7.4)	127	126
4		350 (10.3)	172	178
5		371 (10.9)	183	188
6		403 (11.9)	207	196
7		407 (12.0)	194	213
8		405 (11.9)	187	218
9		390 (11.5)	186	204
10		340 (10.0)	161	179
11		276 (8.1)	139	137
12		204 (6.0)	105	99
Total	(*n*)	3399	1661	1738
	(%)	100.0	48.9	51.1

**Table 2:** Number of participants by region

Region		Number of participants (*n*)			
		Total	(Sampling rate)	Boys	Girls
Hokkaido and Tohoku		504 (14.8)	(0.026)	254	250
Kanto		644 (18.9)	(0.012)	331	313
Chubu		376 (11.1)	(0.014)	174	202
Kinki		691 (20.3)	(0.026)	343	348
Chugoku and Shikoku		441 (13.0)	(0.031)	205	236
Kyushu and Okinawa		743 (21.9)	(0.038)	354	389
Total	(*n*)	3399		1661	1738
	(%)	100	(0.021)	48.9	51.1

Parenthesis; %

**Table 3:** The age-classified distribution of incompetent lip seal prevalence in Japanese children

Age (years)		Number of participants (*n*)	Distribution of ILS prevalence [*n* (%)]*			
			Yes	Think so	Do not think so	No
3		253	18 (7.1)	30 (11.9)	58 (22.9)	147 (58.1)
4		350	24 (6.9)	55 (15.7)	85 (24.3)	186 (53.1)
5		371	34 (9.2)	71 (19.1)	87 (23.5)	179 (48.2)
6		403	28 (6.9)	83 (20.6)	109 (27.0)	183 (45.4)
7		407	35 (8.6)	77 (18.9)	129 (31.7)	166 (40.8)
8		405	45 (11.1)	91 (22.5)	95 (23.5)	174 (43.0)
9		390	36 (9.2)	107 (27.4)	111 (28.5)	136 (34.9)
10		340	48 (14.1)	79 (23.2)	94 (27.6)	119 (35.0)
11		276	36 (13.0)	65 (23.6)	63 (22.8)	112 (40.6)
12		204	28 (13.7)	53 (26.0)	42 (20.6)	81 (39.7)
Total	(*n*)	3399	332 (9.8)	711 (20.9)	873 (25.7)	1483 (43.6)
	(%)	100.0	1043 (30.7)		2356 (69.3)	

*Based on question item 18—“Is your child’s mouth often open during the day?;” *ILS* incompetent lip seal

The prevalence of ILS in six regions throughout Japan is depicted in Table 4. The prevalence of ILS was 30.6% in Hokkaido and Tohoku, 28.6% in Kanto, 30.6% in Chubu, 30.2% in Kinki, 31.7% in Chugoku and Shikoku, and 32.4% in Kyushu and Okinawa. There were no regional differences in ILS in Japan (*p* = 0.506).

**Table 4:** ILS prevalence in Japan, stratified by region

Region		Number of participants (*n*)	Distribution of ILS prevalence [n (%)]*			
			Yes	Think so	Do not think so	No
			“Yes” and “think so”		“No” and “think so”	
Hokkaido and Tohoku		504	57 (11.3)	97 (19.2)	136 (27.0)	214 (42.5)
			154 (30.6)		350 (69.4)	
Kanto		644	59 (9.2)	125 (19.4)	160 (24.8)	300 (46.6)
			184 (28.6)		460 (71.4)	
Chubu		376	31 (8.2)	84 (22.3)	98 (26.1)	163 (43.4)
			115 (30.6)		482 (69.8)	
Kinki		691	64 (9.3)	145 (21.0)	177 (25.6)	305 (44.1)
			209 (30.2)		482 (69.8)	
Chugoku and Shikoku		441	47 (10.7)	93 (21.1)	106 (24.0)	195 (44.2)
			140 (31.7)		301 (68.3)	
Kyushu and Okinawa		743	74 (10.0)	167 (22.5)	196 (26.4)	306 (41.2)
			241 (32.4)		502 (67.6)	
Total	(*n*)	3,399	1,043		2,356	
	(%)	100	30.7		69.3	

*Based on question item 18—“Is your child’s mouth often open during the day?;” *ILS* incompetent lip seal

Table 5 summarizes regional differences in all the survey question items in Japanese children. Item numbers 1, 2, 3, 4, 12, 21, 23, 33, 35, 40, and 41 exhibited regional differences in score, according to the Kruskal-Wallis test (all *p* < 0.05).

**Table 5:** Regional differences in all survey question items

Item no.	Question item	*H*	AP^#^	Item No.	Question item	*H*	AP^#^
1	Does your child get tired easily?	11.355	0.045*	2	Is your child a good riser?	13.791	0.017*
3	Is your child good at exercising?	14.620	0.012*	4	Is your child a restless sleeper?	13.412	0.020*
5	Does your child have round shoulders?	3.494	0.624	6	Does your child’s nose become stuffed easily during the day?	7.065	0.216
7	Does your child’s nose become stuffed easily while sleeping?	0.216	0.216	8	Does your child sneeze often?	2.324	0.803
9	Does your child often have a runny nose?	2.148	0.828	10	Does your child often have a nosebleed?	8.236	0.144
11	Does your child often have a sore throat	6.031	0.303	12	Does your child have swollen tonsils?	22.945	0.000*
13	Does your child often fail to listen?	2.522	0.773	14	Is your child a habitual snorer?	6.885	0.229
15	Is your child’s mouth often dry?	5.323	0.378	16	Do people tell your child that they have bad breath in the morning?	6.054	0.301
17	Do people tell your child that they have bad breath during the day?	9.677	0.085	18	Is your child’s mouth often open during the day?	4.307	0.506
19	Does your child sleep with their mouth open?	3.634	0.603	20	Can your child keep their mouth closed for about 1 minute?	4.464	0.485
21	Does your child have an overbite?	12.040	0.034*	22	Does your child have an underbite?	9.181	0.102
23	Does your child have an anterior open bite?	13.064	0.023*	24	Can your child talk clearly?	4.467	0.484
25	Are your child’s lips often chapped?	2.937	0.710	26	Are your child’s lips thick?	4.200	0.521
27	Is your child’s upper lip turned upward?	9.796	0.081	28	Are your child’s teeth visible between their upper and lower lips?	7.882	0.163
29	Are your child’s lips droopy?	3.990	0.551	30	Are your child’s lips often cracked?	1.746	0.883
31	Are your child’s gums often swollen?	7.572	0.181	32	Are your child’s gums easily stained?	3.519	0.621
33	Are your child’s teeth easily stained?	14.559	0.012*	34	Does your child often have canker sores?	7.823	0.166
35	Does your child have tartar build-up?	20.599	0.001*	36	Do your child’s meals consist of small servings?	3.616	0.606
37	Does your child prefer soft food?	9.534	0.090	38	Does your child drink water during meals?	10.192	0.070
39	Does your child eat fast?	6.508	0.260	40	Is your child a picky eater?	15.559	0.008*
41	Does your child chew food well?	15.879	0.007*	42	Is your child a noisy eater?	7.286	0.200
43	Does your child keep their mouth closed when they eat?	2.597	0.762	44	Does your child have food left in their mouth for a long time?	6.783	0.237

*H* H statistic

^#^Asymptotic significance probability based on a Kruskal-Wallis test is indicated. The degrees of freedom for all items = 5

*p < 0.05

Correlation analysis was conducted via Spearman’s rank correlation coefficient between each question item and ILS status. We observed a relationship (*p* < 0.001) between 12 of the 44 items and ILS status (Table 6). Among these, the question item with the strongest correlation to ILS status was “Are your child’s lips droopy?” (*ρ* = 0.602), with 20.7% of respondents indicating a score of 3 or 4. “Does your child sleep with their mouth open?” (*ρ* = 0.542), “Is your child’s mouth often dry?” (*ρ* = 0.385), and “Are your child’s teeth visible between their upper and lower lips?” (*ρ* = 0.314) also exhibited moderate correlations to an ILS, with 46.0%, 18.7%, and 12.2% of respondents, respectively, indicating a score of 3 or 4. The other eight items exhibited a weak correlation to an ILS (|0.209| ≤ *ρ* ≤ |0.257|). We detected no sex-related differences in any of these items. Using the Cochran-Armitage test for trend, all 12 items exhibited an increase in scores of 3 and 4 with age in Japanese children (*p* < 0.05), and separately in girls (*p* < 0.05). Two of these items did not exhibit a statistically significant increase with age in Japanese boys (*p* > 0.05): “Can your child keep their mouth closed for about 1 minute?” and “Does your child keep their mouth closed when they eat?”

**Table 6:** Correlation and trend with age between 12 question items and an incompetent lip seal

Question item	Total “yes” and “think so” [*n* (%)]	Spearman’s rank correlation coefficient		Cochran-Armitage test for trend with increasing age (*p* value)		
		Correlation	*p* value	Total	Boys	Girls
29. Are your child’s lips droopy?	705 (20.7)	0.602***	< 0.001	< 0.001	< 0.001	< 0.001
19. Does your child sleep with their mouth open?	1565 (46.0)	0.542***	< 0.001	< 0.001	0.003	0.008
15. Is your child’s mouth often dry?	635 (18.7)	0.385***	< 0.001	< 0.001	< 0.001	< 0.001
28. Are your child’s teeth visible between their upper and lower lips?	414 (12.2)	0.314***	< 0.001	< 0.001	< 0.001	0.001
20. Can your child keep their mouth closed for about 1 minute?	297 (8.7)	−0.257***	< 0.001	0.033	0.543	0.012
42. Is your child a noisy eater?	672 (19.8)	0.248***	< 0.001	< 0.001	< 0.001	0.001
7. Does your child’s nose become stuffed easily while sleeping?	957 (28.2)	0.237***	< 0.001	< 0.001	< 0.001	0.006
43. Does your child keep their mouth closed when they eat?	988 (29.1)	−0.232***	< 0.001	0.004	0.087	0.017
21. Does your child have an overbite?	542 (15.9)	0.231***	< 0.001	< 0.001	< 0.001	< 0.001
6. Does your child’s nose become stuffed easily during the day?	1132 (33.3)	0.227***	< 0.001	< 0.001	< 0.001	< 0.001
17. Do people tell your child that they have bad breath during day?	478 (14.1)	0.219***	< 0.001	< 0.001	< 0.001	< 0.001
16. Do people tell your child that they have bad breath in the morning?	1226 (36.1)	0.209***	< 0.001	0.002	0.010	0.022

***p < 0.001

## Discussion

We investigated the prevalence of ILS in 3399 Japanese children and demonstrated an overall ILS prevalence of 30.7%. Gross et al. revealed a prevalence of a resting open-mouth posture of 34.3% in 133 children (8.4 years old), with statistically significant racial differences [20]. Yata et al. also reported the ILS rate of Japanese 7–14-year olds: 45% for those with normal occlusion and 43% for those with malocclusion [28]. De Menezes et al. reported an ILS rate of 34.0% in Brazilian 8–10-year olds, with a statistically significant higher prevalence among mouth breathers than among nasal breathers [29]. In another study of 348 first-grade children, a high proportion (48%) exhibited an open-mouth posture [22]. In the present study, the ILS rate was 33.8% in 8-year olds, consistent with the findings of Gross et al. [19], and 37.4% in 10-year olds, a lower prevalence compared to the findings of Yata et al. [26] for the same age group.

Previous findings raise the question of whether regional factors, such as climate, are important for the development of an ILS. However, there were no regional differences in ILS prevalence in the present study, and to our knowledge, no other reports have described a locational difference in ILS prevalence. Travel and family relocation within the Japanese population may eliminate any potential geographical effects of climate. The major strength of this study is the inclusion of a homogenous study population. A limitation of this study is that factors such as migration within the Japanese population may affect the comparison of regional differences. Inter-prefecture movement was 2.0% in 2019 throughout Japan, a rate that has remained approximately constant for the past two decades [30]. Although age and ethnicity may affect the rate of ILS, globally, 30–50% of children have been reported to have an ILS [6, 8, 16, 20].

We have illustrated that the ratio of ILS statistically significantly increases with age. Notably, the prevalence of scores of 3 or 4 of 12 question items in the survey also exhibited statistically significant increases with age (Table 6). The reason and timing of the acquisition of the ILS in children may vary from person to person as previously described. However, if children acquire an ILS during childhood, the fine balance between the tongue, cheeks, and lips to maintain the teeth and alveolus in the appropriate positions is lost. Moreover, the occlusal relationship and facial soft tissue morphology will gradually worsen with further growth and development [31]. This morphological imbalance may complicate the reversal back to the fine balance. The acquisition of a daily habit to close the lips is particularly important in children. Lip-closing strength related to closed lips increased with age in children, but children with ILS tended to have weak lip-closing strength [11, 32]. Owing to ILS immobilization without spontaneous improvement, the prevalence of ILS may increase with age.

Items, which correlated to an ILS, included “Are your child’s lips droopy?,” “Are your child’s teeth visible between their upper and lower lips?,” and “Does your child have an overbite?” These items are all related to orofacial morphology and are useful for the morphological screening of ILS. The fine balance between the tongue, cheeks, and lips maintains the teeth and alveolus in the appropriate positions [17]. Abnormal oral habits and oral dysfunction may cause an imbalance in oral function, either locally or systemically, during the growth periods [17]. Moreover, Sundelin et al. reported that sleep deprivation may affect the facial appearance, resulting in features such as droopy lips [33]. It has been reported that Japanese children have procured less sleep over the last decade, a phenomenon more pronounced in children living in large cities than in children living in the countryside [34]. However, in this study, Japanese children in the more sparsely populated regions of Hokkaido and Tohoku, Chugoku and Shikoku, and Kyushu and Okinawa tended to be more restless sleepers (question item 4) than the children in the more densely populated areas that included large cities such as Kanto and Kinki; moreover, children in the southern areas tended to be more restless sleepers than those in the northern areas (Table 5). However, restless sleeping was not correlated to the presence of an ILS.

Other question items that correlated with an ILS in this study, namely “Does your child sleep with their mouth open?,” “Can your child keep their mouth closed for about 1 minute?,” and “Is your child’s mouth often dry?,” may be related to mouth breathing [11–13]. Noisy eating and eating with an open mouth both correlated to an ILS in this study and may also be associated with mouth breathing, in which children have trouble with nasal breathing. In a study of Brazilian children aged 8–10 years, 58.8% of mouth breathers had an ILS, compared with 5.7% of nasal breathers [29]. Neiva et al. mentioned a relationship between mouth breathing and restless sleep [35, 36]. Enlargement of the palatine and adenoid tonsils is one of the most common causes of obstructive sleep apnea in children and is most prevalent in patients aged 3–5 years [37, 38]. Hosokawa et al. reported that the tonsil size exhibited a statistically significant increase at 3 years of age, with no statistically significant difference in tonsil size at age 3–12 years [37]. On the other hand, Manabe et al. revealed that the ratio of adenoid and tonsil size to upper airway area exhibited a rapid decrease with age in 8–20-year-old Japanese, following a slow increase between 6 and 8 years of age [39]. Once an ILS becomes a habit, it may continue for a long period despite the resolution of the cause by growth and development. If items of enlargement of the tonsils are detected before the age of 6 years, it may indicate a pharyngeal lymphoid tissue overgrowth. In the present study, however, we discovered no correlation between an ILS and swollen tonsils.

Other question items that correlated to an ILS in this study included a stuffy nose and bad breath, which may be related to allergic rhinitis. “Morning breath” is the common expression for oral malodor at the time of awakening, a common condition with physiological causes. In particular, an irregular daily rhythm (e.g., diet and sleep) is considered as a cause of oral malodor in children since it results in a tongue coating. Kaneita et al. have reported the relationship between insomnia and an irregular daily rhythm in adolescents, suggesting that insomnia may be related to bad breath, and possibly an increase in ILS [40]. Mouth breathing in infants and children is strongly associated with ILS, often an allergic manifestation; therefore, efficient allergy management can forestall the effects of orofacial deformity [41]. This is important, as severe nasal allergy may have the same effect on the orofacial structure as do swollen adenoids [42].

The limitation of our present study is that children under 3 years of age were not included in this study. Although the literacy rate of Japan is 99%, it may be biased depending on the language comprehension of the respondents to the questionnaire. The main strengths of our study are its large sample size and the national scope of the data. We decided that it would be better to collect as many samples as possible for this study since this was the first and most extensive study concerning ILS in children throughout Japan. The external validity in Japanese children can be judged as superior since this national study covered a general population of children from various regions of Japan. The limitations of this study include the cross-sectional nature of data collection and the subjective responses to questions related to ILS.

Dental caries [43, 44] and periodontal disease [34] are two orofacial diseases that receive considerable attention. We believe that ILS should also receive increased attention, as it is prevalent among children, globally and in Japan, and it is related to the factors affecting orofacial diseases.

## Conclusions

In conclusion, we illustrated an ILS rate of 30.7% in Japanese children, which was correlated with self-response survey questions related to orofacial morphology, mouth breathing, and allergic rhinitis. We also elucidated that the rate of ILS increased with age and that there were no regional differences of ILS occurrence in Japanese children. Therefore, this multifactorial disorder appears unlikely to self-correct during growth periods. Pediatric dentists are normally the first oral healthcare professionals to have contact with children with an ILS; therefore, we propose the drafting of guidelines for clinical recognition of ILS among children aged 3–12 years.

## Supplementary Information

**Additional file 1.** Questionnaire on conditions possibly linked to an incompetent lip seal.

## Data Availability

The datasets analyzed during the present study are not publicly available due to ethical restrictions but are available from the corresponding author on reasonable request. All data analyzed during this study are included in this published article.

## Abbreviations

ILS: Incompetent lip seal
