# Supplementary material for: Prevalence of an incompetent lip seal during growth periods throughout Japan: a large-scale, survey-based, cross-sectional study
Source: Environ Health Prev Med. 2021 Jan 21;26:11. doi: 10.1186/s12199-021-00933-5 (PMC7819306; doi:10.1186/s12199-021-00933-5)
Supplement: Supplementary file 1 — Additional file 1. Questionnaire on conditions possibly linked to an incompetent lip seal. [file 12199_2021_933_MOESM1_ESM.docx]

Additional file 1: Questionnaire on conditions possibly linked to an incompetent lip seal

| **Question items**　 Please answer the following questions regarding your child's general and oral condition in daily life.  (For each item: no, do not think so; yes, think so) | 23. Does your child have an anterior open bite?  24. Can your child talk clearly?  25. Are your child’s lips often chapped?  26. Are your child’s lips thick?  27. Is your child’s upper lip turned upward?  28. Are your child’s teeth visible between their upper and lower lips?  29. Are your child’s lips droopy?  30. Are your child’s lips often cracked?  31. Are your child’s gums often swollen?  32. Are your child’s gums easily stained?  33. Are your child’s teeth easily stained?  34. Does your child often have canker sores?  35. Does your child have tartar build-up?  36. Do your child’s meals consist of small servings?  37. Does your child prefer soft food?  38. Does your child drink water during meals?  39. Does your child eat fast?  40. Is your child a picky eater?  41. Does your child chew food well?  42. Is your child a noisy eater?  43. Does your child keep their mouth closed when they eat?  44. Does your child have food left in their mouth for a long time? |
| --- | --- |
|  | 1. Does your child get tired easily?  2. Is your child a good riser?  3. Is your child good at exercising?  4. Is your child a restless sleeper?  5. Does your child have round shoulders?  6. Does your child’s nose become stuffed easily during the day?  7. Does your child’s nose become stuffed easily while sleeping?  8. Does your child sneeze often?  9. Does your child often have a runny nose?  10 Does your child often have a nosebleed?  11. Does your child often have a sore throat?  12. Does your child have swollen tonsils?  13. Does your child often fail to listen?  14. Is your child a habitual snorer?  15. Is your child’s mouth often dry?  16. Do people tell your child that they have bad breath in the morning?  17. Do people tell your child that they have bad breath during the day?  18. Is your child’s mouth often open during the day?  19. Does your child sleep with their mouth open?  20. Can your child keep their mouth closed for about 1 minute?  21. Does your child have an overbite?  22. Does your child have an under bite? |
